# A New and Efficient Method for the Synthesis of Novel 3-Acetyl Coumarins Oxadiazoles Derivatives with Expected Biological Activity

**DOI:** 10.3390/molecules19010911

**Published:** 2014-01-14

**Authors:** Abdullah Sulaiman Al-Ayed, Naceur Hamdi

**Affiliations:** 1 Chemistry Department, College of Science and Arts, Al-Rass, P.O. Box 53, Qassim University, Buraidah 51477, Saudi Arabia; 2 Higher Institute of Environmental Science and Technologies (HIEST-Borj Cedria, Tunisia), University of Carthage, Touristic Road of Soliman, BP 95, Hammam-Lif 2050, Tunisia

**Keywords:** radical scavenging activity, antibacterial, antifungal, antioxydant, coumarin

## Abstract

This paper presents the design of some novel 3-acetylcoumarin derivatives, based on minimal inhibitory concentration values (MICs) previously obtained against some microorganism cultures, Gram positive and negative bacteria and fungi. Some of these molecules exhibited antibacterial activity against *S.* *aureus*, comparable to that of the standard used (impinem). The *in* *vitro* antioxidant activities of the novel 3-acetylcoumarin oxadiazoles were assayed by the quantitative 1,1-diphenyl-2-picrylhydrazyl (DPPH) radical scavenging activity method. The compounds **5c**,**d** proved to be the most active, showing the highest capacity to deplete the DPPH radicals. Structure elucidation of the products has been accomplished on the basis of IR, ^1^H-NMR, ^13^C-NMR, NOESY and HMBC NMR data.

## 1. Introduction

Due to the interesting activity of 2,5-disubstitued 1,3,4-oxadiazoles as biological agents considerable attention has been focused on this class of heterocycles. The pharmaceutical importance of these compounds lies in the fact that they can be effectively utilized as antioxidant, antibacterial, antitubercular and insecticidal agents [1,2,3,4]. Some of these compounds have also analgesic, anti-inflammatory, anticancer, anti-HIV agent and antiproliferative agent activity [5,6,7,8,9,10,11,12,13].

Coumarin and its derivatives represent one of the most active classes of compound possessing a wide spectrum of biological activity [14,15,16,17,18,19,20,21,22,23,24,25,26,27,28,29,30,31]. Many of these compounds have proven to be active as antimicrobial [32], anti-inflammatory [33], and antitumor [34,35] agents and in inhibition of hepatitis C virus [36,37,38], however, their natural abundance in plants is very low and the purification processes are complex. Therefore, various methods have been developed for the total synthesis of coumarins [39,40,41], including the Perkin [42], Pechmann [43], and Knoevenagel reactions [44]. Herein, we report the highly efficient total synthesis of five novel 3-acetylcoumarin oxadiazole derivatives. To the best of our knowledge, this is the first report on the total synthesis of these compounds. The preparation of 3-acetyl ethyl(coumarin-4-oxy) acetate (**2**) which was used as a starting material for the synthesis of compounds **5**, is also presented in this study. The chemical structures of the synthesized compounds **5** were determined by spectroscopic techniques and confirmed. Their antibacterial, antifungal, and antioxidants activities were investigated.

## 2. Results and Discussion

**Scheme 1:**
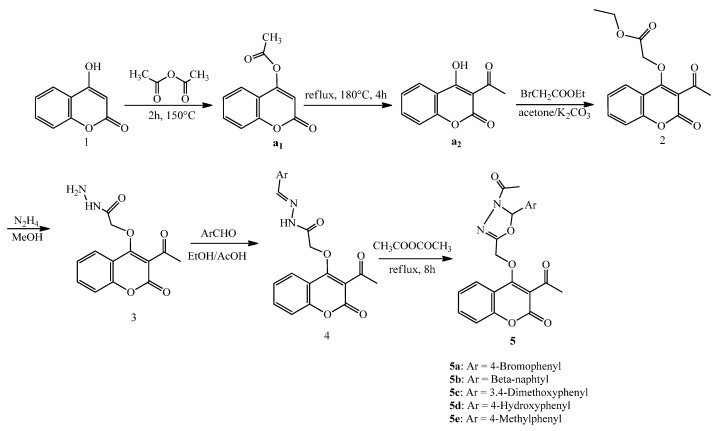
Synthesis of 3-acetylcoumarin derivatives **5**.

The synthesis of the target compounds was carried out according to the representative Scheme 1. The starting material 4‑hydroxycoumarin was reacted with acetic anhydride to produce the corresponding *O*-acyloxyl product **a_1_**.

It was observed that a proton on the hydroxyl group of coumarin was substituted by an acyl group when we elevated the temperature to *ca.* 150 °C. The subsequent reaction involving heating to over 170 °C in a sealed tube led to the rearrangement product **a_2_** in yields comparable to those of **a_1_**. Compound **2** was prepared in 92% yield by refluxing ethyl bromoacetate with the O-acyloxyl product **a_2_** in anhydrous acetone in the presence of anhydrous potassium carbonate.

Hydrazinolysis of **2** with 86% hydrazine hydrate in methanol at room temperature afforded hydrazide **3** in good yield. The FT-IR spectrum of compound **3** showed absorption bands in the 3293.3–3211.0 cm^−1^ (hydrazide NH-NH_2_) and 1715 cm^−1^ (lactonic-C=O carbonyl stretching). The ^1^H-NMR spectrum exhibited a singlet due to the –NH proton at δ 8.89 ppm. The –OCH_2_methylene protons resonated as a singlet at 4.89 ppm. Boiling hydrazide **3** with different aryl aldehydes in absolute ethanol in the presence of a catalytic amount of glacial acetic acid for 2 to 4 h afforded the corresponding compounds **4**. The thus obtained compounds **4** were then refluxed with acetic anhydride for 6–8 h to give the corresponding substituted 3-acetylcoumarin oxadiazoles **5** in good yields.

All the new substituted oxadiazoles **5** have been characterized by IR, ^1^H-NMR, ^13^C-NMR spectroscopy, as well as by NOESY and HMBC NMR experiments to elucidate their structures and completely assign the structural network of both protons and carbons. The IR spectra of compound **5c**, for example, showed characteristic bands at 1612 cm^−^^1^ (C=C), 1289 cm^−^^1^ (lactone C-O-C) and 1556 cm^−^^1^ (N=N). Its ^1^H-NMR spectrum showed the disappearance of the NH_2_ protons at 3.38, and instead, it displayed a signal at 8.30 ppm for the N=CH proton at 8.30 ppm. The singlet signal at δ 6.07 ppm was assigned to the H-3 proton. The methyl protons of the acyl group appear as a singlet at δ 2.38 ppm, while the H-5' proton from the oxodiazole fragment appeared as a singlet at δ 8.53 ppm. The aromatic protons (both coumarinic and oxodiazolinic) are observed between δ 7.08 and δ 7.91 ppm. The structure of compounds **5** was also established by ^1^H-^1^3C HMBC 2D NMR spectroscopy, which shows the C_5_–C_4_–C_3_–Me linkages. A correlation between protons (b) and carbons C_2_ and C_4_ at 162.0 ppm 164.8 ppm, respectively, was observed. Another correlation was also observed between the H_5_ proton and an aromatic carbon at 125.5 ppm.

### 2.1. Antibacterial Studies

**Figure 1:**
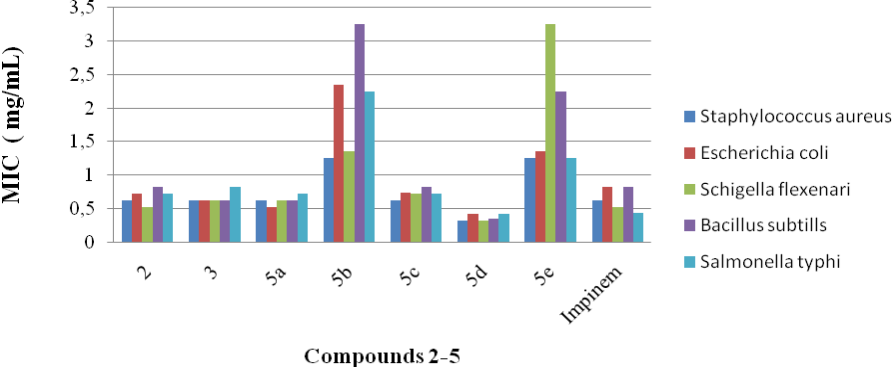
MIC value of the synthesized compounds **2**–**5** in mg/mL.

The synthesized compounds **2**–**5** were checked against various microorganisms such as *Salmonella* *typhi*, *Schigella* *flexenari*, *Escherichia* *coli*, *Staphylococcus* *aureus* and *Bacillus* *subtills* in order to establish their bioactivities. In these tests impinem was used as the standard drug [45]. The results obtained against these microorganisms are given in Figure 1.

The results obtained clearly show the efficiency of some of the new compounds, even at low concentrations. The results indicated that some of the synthesized compounds have higher activity than the standard.

We found that the activity of the synthesized compounds depends on their concentration and the strain of tested bacteria. Gram positive bacteria were more susceptible to the synthesized compounds than Gram negative ones. This effect can be attributed in part to the great complexity of the double membrane-containing cell envelope in Gram negative bacteria, compared to the single membrane structure of positive ones.

### 2.2. Statistical Analysis

Results were expressed as the mean ± standard error means (S.E.M.). The comparison of data within groups was performed by the analysis of variance using ANOVA test. A probability level of less than 1% (*p* < 0.01) was considered significant. The statistical analysis was made by using Systat 7.0.

### 2.3. Antifungal Activity

**Figure 2:**
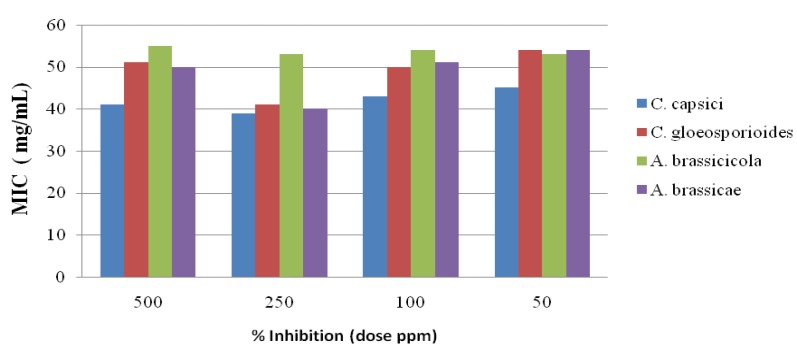
Antifungal results for compound **5a**.

**Figure 3:**
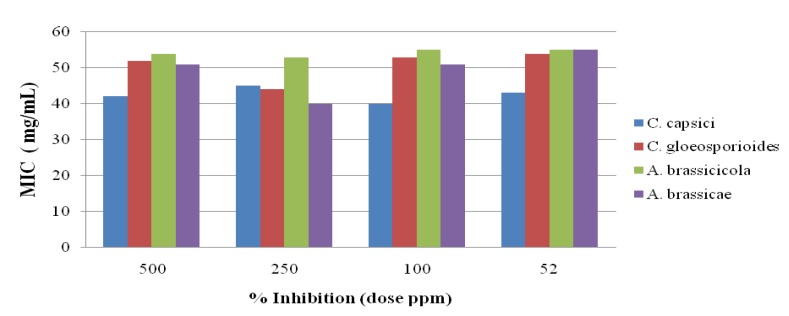
Antifungal results for compound **5b**.

**Figure 4:**
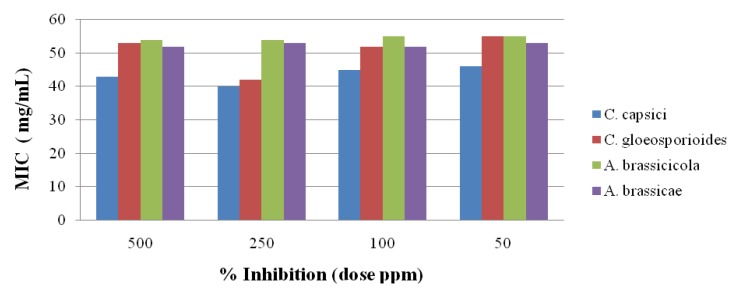
Antifungal results for compound **5c**.

**Figure 5:**
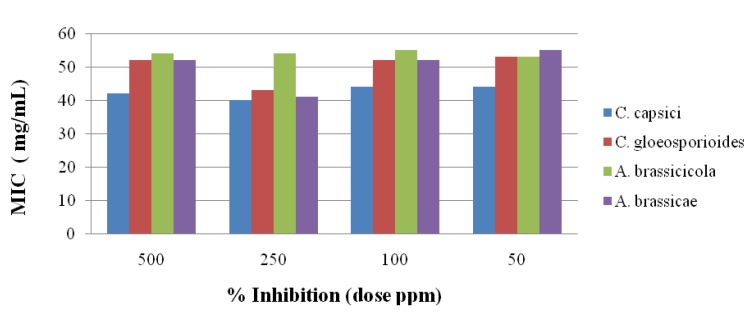
Antifungal results for compound **5d**.

**Figure 6:**
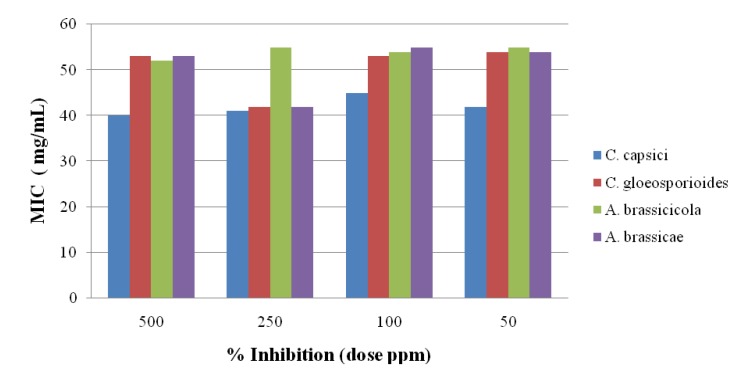
Antifungal results for compound **5e**.

The antifungal activity of the newly synthesized oxadiazole **5** derivatives has been studied against the *C. capsici*, *C. gloeosporioides*, *A. brassicicola* and *A. brassicae*. Fluconazole [46] and DMSO were used as positive and negative controls, respectively. The antifungal study results are shown in Figure 2, Figure 3, Figure 4, Figure 5 and Figure 6. The results showed that all the compounds possess excellent fungal toxicity.

It has also been observed that toxicity is enhanced as the concentration of compounds increases. Compound **5d** showed promising activity against all fungi at a dose of 500 ppm and even at lower concentration (Figure 2). Compounds **5b**,**c** exhibit moderate activity at all concentrations (Figure 3, Figure 4, Figure 5 and Figure 6).

Positive control: fluconazole; negative control: DMSO.

Positive control: fluconazole; negative control: DMSO.

Positive control: fluconazole; negative control: DMSO.

Positive control: fluconazole; negative control: DMSO.

Positive control: fluconazole; negative control: DMSO.

### 2.4. Antioxidant Activities

**Figure 7:**
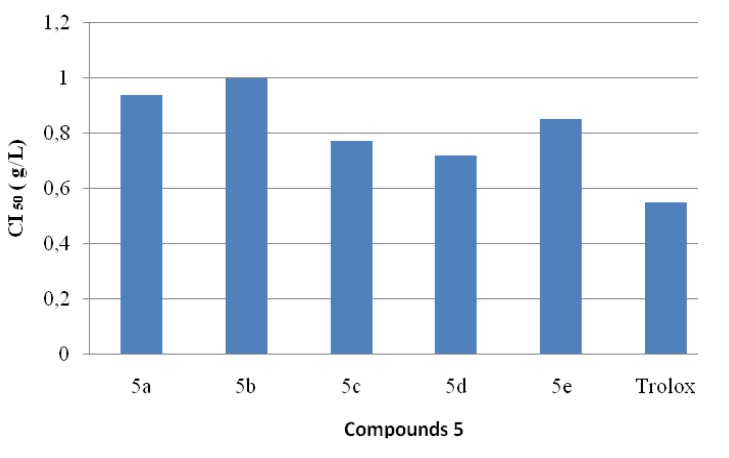
.Values of IC_50_ exhibited by coumarinic derivatives **5a**–**e**.

The antiradical activities of antioxidants can be determined using the free radical, 2,2-diphenyl-1-picrylhydrazyl (DPPH*). In its radical form, DPPH* has an absorption band at 515 nm which disappears upon reduction by an antiradical compound. Compounds **5** were thus screened for *in* *vitro* antioxidant activity using the DPPH* free radical assay and the results are shown in Figure 7. The effect of the different synthetic compounds on DPPH radical scavenging was compared to those of Trolox [47], used as positive control, and appreciated by the determination of the IC_50_ values.

The data proved that compound **5d** showed the strongest antioxidant activity.

### 2.5. ABTS Radical Cation Decolourization Assay

**Figure 8:**
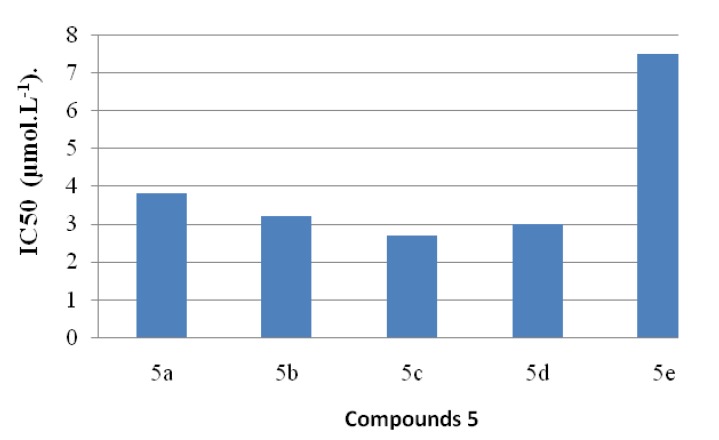
Scavenging ability on ABTS radical of compounds **5**.

As shown for DPPH scavenging, the obtained results indicate the higher capacity of **5a**–**d** to quench ABTS as compared to the synthetic antioxidant Trolox (Figure 8).

## 3. Experimental

### 3.1. General Information

Melting points were determined on a Köfler block and are uncorrected. IR spectra were recorded on a Perkin-Elmer 983 IR spectrophotometer (Perkin-Elmer, Waltham, MD, USA) as KBr pellets. The ^1^H-NMR spectra were recorded on a Bruker AC 300F (300 MHz) (Bruker, Rheinstetten, Germany) using DMSO-*d_6_* or CDCl_3_ as solvents and TMS as internal standard. NMR assignments were obtained from examination of 1D and 2D experiments (^1^H, ^13^C, NOESY and HMBC). Mass spectra were recorded on a GC-MS solution DI Analysis Shimadzu Qp-2010 unit (Shimadzu, Kyoto, Japan). Elemental analysis was determined at the University of Rennes 1, Rennes, France. Thin layer chromatography (TLC) was carried out on silica gel plates (particle size 0.030–0.040 mm, pore diameter ca. 6 nm). Solutions were evaporated under diminished pressure unless otherwise stated. The ChemDrew-Ultra-8.0 software was used for naming the prepared compounds.

### 3.2. Preparation of 4-O-Acetylcoumarin (**a**_1_) and 3-Acetyl-4-hydroxycoumarin (**a**_2_)

4-Hydroxycoumarin (**1**, 0.01 mol) was added to a high-pressure tube containing acid anhydride (5 mL). The solution was stirred for 2 h at 150 °C. The title compound was separated by column chromatography (CHCl_3_/ethyl acetate/*n*-hexane = 8:1:1). Next the solution was stirred at 180 °C and then purified by column chromatography to obtain the rearranged compound **a_2_**.

**a_1_**. M.p.: 128–130 °C. ^1^H-NMR (CDCl_3_) δ: 6.32 (1H, s), 7.26 (1H, d, *J* = 7.5 Hz), 7.46 (1H, dd, *J* = 7.5, 2.1 Hz), IR (KBr, cm^−1^) 1,645, 1,680 (C=O).

**a_2_**. M.p.: 194–196 °C. ^1^H-NMR (CDCl_3_) δ: 2.73 (3H, s), 3.89 (3H, s), 6.72 (1H, d, *J* = 2.1 Hz), 6.85 (1H, dd, *J* = 8.9, 2.1 Hz), 7.92 (1H, d, *J* = 8.9 Hz). IR (KBr, cm^−1^) 1661, 1753 (C=O), 3442 (OH). LC/MS/MS (ESI) *m/z*, (rel. int. %): 234.9 (M^+^, 20), 217.3 (M^+^–OH, 100), 192.9 (M^+^‒acetyl, 62), 151.1 (74).

### 3.3. Preparation of Compounds **2–5**

#### 3.3.1. Preparation of 3-Acetyl Ethyl (coumarin-4-oxy) Acetate (**2**)

A mixture of 3-acetyl-4-hydroxycoumarin (**a_2_**, 0.1 mol), anhydrous potassium carbonate (0.1 mol), and ethyl bromoacetate (0.1 mol) was refluxed in dry acetone (10 mL) for 10 h. The resulting solution was left at R.T., and the product that separated out was filtered off and washed with acetone. Yield: 80%; M.p.: 245 °C. IR (υ, cm^–1^): 1732 (CO); 1525 (O-CO lactone). ^1^H-NMR (DMSO-*d_6_*) δ: 1.23 (t, 3H, CH_3_,* J* = 7.9 Hz), 2.26 (s, 3H, CH_3_), 4.23 (q, 2H, CH_2_,* J* = 7.9 Hz), 5.1 (s, 2H, OCH_2_), 5.93 (s, 1H, H_3_), 7.36–7.82 (m, 4H, H_arom_). ^13^C-NMR spectrum (DMSO-*d_6_*) δ: 14.2 (CH_3_), 61.4 (CO-O-CH_2_), 91.7 (C_3_), 65.7 (OCH_2_), 115.2–153.0 (C_arom_), 161.7 (C_2_), 164.3 (C_4_), 167.3 (CO_ester_), 172.5 (CO).

#### 3.3.2. Preparation of 3-Acetylcoumarin-4-oxyacetic Hydrazide (**3**)

A solution of compound **2** (0.037 mol) and hydrazine hydrate (0.022 mol) in methanol (25 mL) was stirred for 7 h at room temperature. After concentrating the reaction mixture a solid mass separated out and was recrystallized using ethanol to give a white precipitate of 3-acetylcoumarin-4-oxyacetic hydrazide (**3**) which was crystallized to give colourless plates. Yield: 85%; M.p.: 270 °C. IR (υ cm^–1^): 1720 (CO); 1715 (O–CO lactone), 3293.3–3211.0 (–NHNH_2_). ^1^H-NMR (DMSO-*d_6_*) δ ppm: 5.70 (s, 3H, H_3_), 4.78 (s, 2H, OCH_2_), 4.43 (s, 2H, NH_2_), 7.34–8.03 (m, 4H, H_arom_), 8.53 (s, 1H, NH). ^13^C-NMR (DMSO-*d_6_*) δ ppm: 67.4 (OCH_2_), 91.6 (C_3_), 115.3–133.2 (C_arom_), 161.8 (C_2_), 164.8 (C_4_), 165.3 (CONH), 173.5 (CO).

#### 3.3.3. Preparation of 3-Acetyl-(*E*)-*N*-arylidenecoumarin-4-oxyacetic Hydrazones **4**

A mixture of hydrazide **3** (0.01 mol) and the appropriate aldehyde (0.01 mol) was refluxed for 5 h in a methanol–water mixture (20 mL). The reaction mixture was cooled and the separated product was filtered off, dried and recrystallized using ethanol.

*3-Acetyl*-*(E)-N-(4-bromobenzylidene)* *coumarin-4-oxyacetic* *hydrazone* (**4a**). Yield: 75%; M.p.: 236 °C. IR (υ cm^–1^): 1694 (C=O); 1435 (C=N); 3120 (NH). ^1^H-NMR (DMSO-*d_6_*) δ ppm: 5.86 (s, 1H, H_3_), 5.56 (s, 2H, OCH_2_), 11.82 (s, 1H, NH), 7.12–8.02 (m, 8H, H_arom_), 5.95 (s, 1H, N=CH). ^13^C-NMR (DMSO-*d_6_*) δ ppm: 66.4 (OCH_2_), 91.6 (C_3_), 115.6–133.3 (C_arom_), 147.5 (N=C), 161.9 (C_2_), 165.1 (C_4_), 167.6 (C_1__'_), 166.3 (CONH), 172.5 (CO). Anal. Calcd for C_18_H_13_N_2_O_4_Br: C, 55.04; H, 3.96; Br, 17.44; N, 6.11; O, 17. Found C, 56.1; Br, 17.6; H, 3.97; N, 5.8.

*3-Acetyl-(E)-N-(β-naphtyl)coumarin-4-oxyacetic* *hydrazone* (**4b**). Yield: 80%; M.p.: 226 °C. IR (υ cm^–1^): 1692 (C=O), 1410 (C=N), 3120 (NH). ^1^H-NMR (DMSO-*d_6_*) δ ppm: 5.54 (s, 2H, OCH_2_), 5.88 (s, 1H, H_3_), 7.02–7.94 (m, 11H, H_arom_); 7.97 (s, 1H, N=CH), 11.85 (s, 1H, NH). ^13^C-NMR (DMSO-*d_6_*) δ ppm: 56.5 (OCH_3_), 60.7 (OCH_3_), 66.6 (OCH_2_), 91.7 (C_3_), 104.7–144.5 (C_arom_), 144.6 (NC), 162.8(C_2_), 165.2 (C_4_), 167.6 (C_1__'_), 167.3 (CONH), 173.5 (CO). Anal. Calcd for C_22_H_18_N_2_O6: C, 69.92; H, 4.93; N, 6.52; O, 18.63 Found C, 69.8; O, 17.6; H, 4.95; N, 6.5.

*3-Acetyl-(E)-N-(3,4-dimethoxybenzylidene)coumarin-4-oxyacetic* *hydrazone* (**4c**). Yield: 80%; M.p.: 226 °C. IR (υ cm^–1^): 1692 (C=O), 1410 (C=N) 3125 (NH). ^1^H-NMR (DMSO-*d_6_*) δ ppm: 3.72 (s, 3H, OCH_3_), 3.84 (s, 3H, OCH_3_), 5.54 (s, 2H, OCH_2_), 5.86 (s, 1H, H_3_), 7.12–7.95 (m, 6H, H_arom_), 7.98 (s, 1H, N=CH), 11.84 (s, 1H, NH). ^13^C-NMR (DMSO-*d_6_*) δ ppm: 56.5 (OCH_3_), 60.8 (OCH_3_), 66.4 (OCH_2_), 91.7 (C_3_), 105.7–144.6 (C_arom_), 144.6 (NC), 162.8 (C_2_), 165.3 (C_4_), 167.7 (C_1__'_), 165.3 (CONH), 173.5 (CO). Anal. Calcd for C_20_H_18_N_2_O_6_: C, 62.86; H, 5.28; N, 6.37; O, 25.49 Found C, 62.8; O, 25.4; H, 5.20; N, 6.3.

*3-Acetyl*-*(E)-N-(4-hydroxybenzylidene)coumarin-4-oxyacetic* *hydrazone* (**4d**). Yield: 75%; M.p.: 248 °C. IR (υ cm^–1^): 1715 (C=O), 1629 (C=N), 2963 (NH). ^1^H-NMR (DMSO-*d_6_*) δ ppm: 5.97 (s, 1H, H_3_), 5.73 (s, 1H, OH), 5.50 (s, 2H, OCH_2_), 8.25 (s, 1H, N=CH), 11.86 (s, 1H, NH), 7.40–8.28 (m, 8H, H_arom_). ^13^C-NMR (DMSO-*d*_6_) δ ppm: 66.5 (OCH_2_); 91.9 (C_3_), 116.2–134.3 (C_arom_), 145.2 (N=C), 162.9 (C_2_), 166.1 (C_4_), 168.4 (C_1__'_), 166.3 (CONH), 171.5 (CO) Anal. Calcd for C_18_H_18_N_2_O_5_: C, 63.79; H, 4.84; N, 7.09; O, 24.28 Found C, 63.7; O, 24.2; H, 4.80; N, 6.9.

*3-Acetyl*-*(E)-N-(4-methylbenzylidene)coumarin-4-oxyacetic* *hydrazine* (**4e**). Yield: 73%; M.p.: 265 °C. IR (υ cm^–1^): 1685 (C=O), 1687 (C=N), 3107 (NH). ^1^H-NMR (DMSO-*d_6_*) δ ppm: 2.80 (s, 3H, CH_3_), 4.96 (s, 1H, H_3_), 5.46 (s, 2H, OCH_2_), 5.92 (s, 1H, N=CH), 11.67 (s, 1H, NH), 6.98–8.30 (m, 8H, H_arom_). ^13^C-NMR (DMSO-*d_6_*). δ ppm: 55.70 (OCH_3_), 66.5 (OCH_2_), 91.8 (C_3_), 114.7–133.4 (C_arom_), 144.6 (N=C), 162.8 (C_2_), 166.3 (CONH), 172.5 (CO), 167.5 (C_1__'_). Anal. Calcd for C_19_H_16_N_2_O_4_: C, 67.16; H, 5.38; N, 7.12; O, 20.33 Found C, 67.1; O, 20.5; H, 3.8; N, 7.1.

#### 3.3.4. Preparation of 3-Acetyl-2-[(coumarin-4-oxy)methyl]-4-acetyl-5-substitued-1,3,4-oxodiazolines **5**

A mixture of compound **4** (0.001 mol) and acetic anhydride (10 mL) was refluxed for 8 h. The hot solution was poured onto ice water (10 mL) and the product which separated was filtered off, washed several times with water, recrystallized from ethanol and dried.

*3-Acetyl-4-(4-acetyl-5-(4-bromophenyl)-4,5-dihydro-1,3,4-oxodiazol-2-yl)methoxy)-2H-chromen-2-one* (**5a**). Yield: 75%, M.p.: 232 °C. IR (υ cm^–1^): 1632 (C=N), 1407 (N–N), 1247 (C–O–C). ^1^H-NMR (DMSO-*d_6_*) δ ppm: 2.42 (s, 3H, CH_3_), 5.49 (s, 2H, OCH_2_), 6.1 (s, 1H, H_3_), 7.35–7.99 (m, 8H, H_arom_), 5.68 (s, 1H, H5'). ^13^C-NMR (DMSO-*d_6_*) δ ppm: 26.2 (CH_3_), 69.4 (OCH_2_), 91.8 (C_3_), 115.6 (C_5__'_), 116.5–133.3 (C_arom_), 162.5 (C_2_), 164.9 (C_4_), 166.7 (C=O), 171.3(C_2__'_), 166.3(CO). Anal. Calcd for C_22_H_17_N_2_O_6_Br: C, 55.21; H, 4.03; Br, 15.97; N, 5.60; O, 19.19 Found C, 56.1; Br, 15.9; O, 19.2; H, 3.98; N, 5.8.

*3-Acetyl-(4-acetyl-5-(β*-*naphtyl)-4,5-dihydro-1,3,4-oxodiazol-2-yl)methoxy)-2H-chromen-2-one* (**5b**). Yield: 76%; M.p.: 248 °C. IR (υ cm^–1^): 1635 (C=N), 1409 (N–N), 1285 (C–O–C). ^1^H-NMR (DMSO-*d_6_*) δ ppm: 2.43 (s, 3H, CH_3_), 4.96 (s, 2H, OCH_2_), 5.86 (s, 1H, H3), 7.21–7.77 (m, 11H, H_arom_), 5.35 (s, 1H, H_5__'_). ^13^C-NMR (DMSO-*d_6_*) δ ppm: 20.6 (CH_3_), 57.3 (OCH_3_), 56.5 (OCH_3_), 60.8 (OCH_2_), 91.3 (C_3_), 116.7 (C_5__'_), 123.4–153.8 (C_arom_), 162.5 (C_2_), 164.6 (C_4_), 173.4 (CO), 193.2 (C_2__'_), 167.3 (CO). Anal. Calcd for C_26_H_20_N_2_O_6_: C, 68.78; H, 4.92; N, 5.94; O, 20.36 Found C, 68.8; O, 20.6; H, 4.85; N, 5.8.

*3-Acetyl-(4-acetyl-5-(3,4-dimethoxyphenyl)-4,5-dihydro-1,3,4-oxodiazol-2-yl)methoxy)-2H-chromen-2-one* (**5c**): Yield: 75%; M.p.: 250 °C. IR (υ cm^–1^) 1635 (C=N), 1410 (N–N), 1285 (C–O–C). ^1^H-NMR (DMSO-*d_6_*) δ ppm: 2.42 (s, 3H, CH_3_), 3.77 (s, 3H, OCH_3_), 3.80 (s, 3H, OCH_3_), 4.85 (s, 2H, OCH_2_), 5.86 (s, 1H, H_3_), 7.22–7.57 (m, 6H, H_arom_), 5.44 (s, 1H, H_5’_). ^13^C-NMR (DMSO-*d_6_*) δ ppm: 20.8 (CH_3_), 56.5 (OCH_3_), 56.4 (OCH_3_), 60.7 (OCH_2_), 92.9 (C3), 116.7 (C5'), 124.3–155.7 (C_arom_), 162.5 (C_2_), 164.9 (C_4_), 172.5 (CO), 192.3 (C_2_), 166.5 (CO). Anal. Calcd for C_24_H_22_N_2_O_8_: C, 62.36; H, 5.23; N, 5.82; O, 26.58 Found C, 62.3; O, 26.6; H, 5.2; N, 5.8.

*3-Acetyl-4-(4-acetyl-5-(4-hydroxyphenyl)-4,5-dihydro-1,3,4-oxodiazol-2-yl)methoxy)-2H-chromen-2-one* (**5d**). Yield: 76%. M.p.: 226 °C. IR (υ cm^–1^): 1628 (C=N), 1415 (N–N), 1243 (C–O–C). ^1^H-NMR (DMSO-*d_6_*) δ ppm: 2.28 (s, 3H, CH_3_), 5.69 (s, 1H, OH), 5.42 (s, 2H, OCH_2_), 5.94 (s, 1H, H3), 7.28–8.29 (m, 8H, H_arom_), 5.82 (s, 1H, H_5__'_). ^13^C-NMR (DMSO-*d_6_*) δ ppm: 20.7 (CH_3_), 66.5 (OCH_2_), 91.9 (C_3_), 115.5 (C_5__'_), 116.4–153.1 (C_arom_), 157.5 (C_2_), 165.8 (C_4_), 163.6 (CO), 164.8 (C_2__'_), 165.3 (CO). Anal. Calcd for C_22_H_18_N_2_O_7_: C, 63.15; H, 4.84; N, 6.40; O, 25.60 Found C, 63.1; O, 25.6; H, 4.9; N, 6.4.

*3-Acetyl-4-(4-acetyl-5-(4-methylphenyl)-4,5-dihydro-1,3,4-oxodiazol-2-yl)methoxy)-2H-chromen-2-one* (**5e**). Yield: 77%; M.p.: 268 °C. IR (υ cm^–1^): 1625 (C=N), 1385 (N–N), 1247 (C–O–C). ^1^H-NMR (DMSO-*d_6_*) δ ppm: 2.28 (s, 3H, CH_3_), 2.36 (s, 3H, CH_3_), 5.36 (s, 2H, OCH_2_), 6.2 (s, 1H, H3), 7.17–7.85 (m, 8H, H_arom_), 5.56 (s, 1H, H5’). ^13^C-NMR (DMSO-*d_6_*) δ ppm: 25.8 (CH_3_), 24.6 (CH_3_), 55.8 (OCH_3_), 69.4 (OCH_2_), 91.8 (C3), 114.2 (C_5__'_), 116.5–153.1 (C_arom_), 164.0 (C_2_), 165.8 (C_4_), 167.0 (CO), 173.2 (C_2__'_), 167.3 (CO). Anal. Calcd for C_23_H_20_N_2_O_6_: C, 66.20; H, 5.32; N, 6.43; O, 22.05 Found C, 66.1; O, 22.1; H, 5.3; N, 6.5.

### 3.4. Antioxidant Activity Tests

#### 3.4.1. Free Radical Scavenging Activity Evaluation by DPPH Test

The DPPH free radical scavenging activity measurements were carried out according to the procedure of Sanchez-Moreno *et* *al.* [48] with some modifications. Briefly, the solution of compound **5** (0.1 mL) was added to 1,1-diphenyl-l-2-picrylhydrazyl radical (DPPH*; 2.46 mL, 0.025 g·L^−1^ in 50% ethanol) and mixed for 5 min. The absorbance of the samples was measured at 515 nm every 1 min for 5 min using a Genesys 2 spectrophotometer (Genesys 10uv scanning, Thermo Spectronic, Rochester, NY, USA). For each sample, three separate determinations were carried out. The antioxidant activity was expressed as the percentage of decline of the absorbance after 1 min, relative to the control, corresponding to the percentage of DPPH*d that was scavenged. The percentage of DPPH*d, which was scavenged (% DPPH sc) was calculated using the following formula:

DPPH scavenging effect (%) = (A_control_ − A_sample_/A_control_) × 100 (1)

where A_control_ is the absorbance of the control reaction were the sample is replaced by 500 µL ethanol. Tests were carried out in triplicate.

#### 3.4.2. Free Radical Scavenging Activity Evaluation by ABTS Radical Cation Test

ABTS radical cation (ABTS^+·^) was produced by reacting ABTS stock solution with 2.45 mM potassium persulfate and allowing the mixture to stand in the dark at room temperature for 24 h before use 10. Afterwards, the ABTS^+·^ solution was diluted with ethanol to an absorbance of 0.7 at 734 nm. After addition of diluted ABTS^+·^ solution (990 mL) to sample (10 mL), absorbance readings were taken at 30 °C exactly 6 min after initial mixing, at 734 nm, using a Genesys 2 spectrophotometer. Absorption of a blank sample containing the same amount of ethanol and ABTS^+·^ solution acted as negative control.

## 4. Conclusions

In this study, an expeditious, atom economic methodology for the synthesis of novel 3-acetylcoumarin oxadiazoles has been described, and the products characterized by IR/1D/2D NMR and IR spectroscopy The antioxidant activity of the novel 3-acetylcoumarin oxadiazoles was tested and they displayed improved properties compared to Trolox. The synthesized compounds **5** were tested for antifungal activities and the results indicated significant activities as compared with fluconazole. The antimicrobial tests performed on coumarin derivatives **5** confirmed the better potential activities of these compounds against Gram-positive rather than Gram-negative bacteria.
